# Propriety of Operating in Cases of Cataract

**Published:** 1844-03

**Authors:** 


*Propriety of operating in cases of Cataract, where only one Eye is Affected.*—Mr. Nunneley, of Leeds, has discussed this subject in a paper read before the *Prov. Med. and Surg. Association*, and published in the *Provincial Journal*, Sept., 1843. Mr. N. conceives that the propriety of operating, or not, must be mainly determined by "the state of vision after the operation," for he remarks: "I suppose, although it be granted a person sees sufficiently well with one eye, no one will deny that *cæteris paribus*, two eyes are better than one; and from the well known fact that when the function of any organ or structure is long suspended, the power of exercising the function becomes ultimately lost, it is, as a mere result of precaution, extremely important to keep the affected eye in such a state of activity, that in case any accident or disease happen to the other, its powers, even though somewhat impaired, may then be taken advantage of; which can only be safely and effectually secured by having removed the opaque crystalline lens, and permitted the light to keep up the activity of the retina. The fear of the sound eye being injured or lost by the operation upon the cataractous one, though possible, is, I I consider, hardly deserving of notice; because, when the operation is properly performed, it must be so rare, as to be rather amongst possibilities than the probabilities. While, on the other hand, the sympathy between the two eyes, not only in their healthy states, but in their morbid condition, is so strong that those who have been accustomed to watch ophthalmic affections will at once admit the validity of the argument of removing any diseased condition of one eye lest the other also partake of it, for the singularity is, that when disease exists in one eye, not only is the other apt to become impaired, but for the corresponding structure to assume the very same morbid condition. Thus, if the conjunctiva in one is affected, that of the other is also very apt to assume the same diseased action; if the cornea, the cornea; if the iris, the iris; the lens, the lens; and so on; while it is also incontrovertible that the morbid condition of the eye primarily affected being removed, that of the one sympathetically or secondarily involved is also frequently remedied. Indeed, cases are on record where cataract having been removed in one eye, commencing cataract, or even amaurosis, in the other has been cured; and there must have been many, if not identical, at least analogous, instances. Indeed, in some cases where I have operated upon an eye in which cataract was fully formed, being only in an incipient state in the other, I have strongly suspected the progress of this latter has been much delayed by the removal of the opaque lens of the opposite eye.

"Now, although every one may not be inclined to think the remote risk of the sound eye being lost from injury or accidental affection very great, nor the danger of sympathetic disease so imminent as to justify our incurring any immediate hazard to it by operative interference with the affected eye, yet no person will deny, that if in reality there is no such danger to the sound eye by operating upon the affected one, the possibility of these remote contingencies are solid arguments in favor of active measures being at once resorted to. The last argument, of confusedness of vision being the result of an operation, is so very plausible, and, indeed, imposing, that it is this which has, I presume, principally determined the general practice of not interfering when only one eye is affected, and which, I confess, formerly decided my practice; for I have sent many persons away without doing anything which, with what I have since seen, especially in the three cases to which I shall now shortly allude, I should certainly not do. Indeed, the fact itself that traumatic cataract sometimes disappears, as mentioned by Pott and Hey, as an argument against operating, is, in reality, a strong argument in favor of it; for, if not all, at least in such of those cases where traumatic cataract disappears, the capsule of the lens has been ruptured, and subsequently has been absorbed, thus occurring what it is the object of the operation to accomplish; yet in these cases no mention is made of inconveniences resulting from the cure; and when the lens had been so displaced as to press upon the iris, everybody agrees as to the necessity of manual interference, lest not only the one eye be altogether lost, but the other be implicated in the change."

Mr. N. relates three cases to show that "in point of fact, the double confusedness of vision, so much feared, does not occur," and concludes that "considering, on the one hand, the ease with which the operation may be performed; the little or no disturbance produced, either to the other eye or general health; that in many cases of traumatic cataract, where the capsule is ruptured, the lens is ultimately removed, even when the surgeon does not interfere; and that what has been so much feared, and in my opinion constituted the only valid argument against the operation, the difference in the refractive powers of the two eyes producing confused or double vision, in reality does not occur; and, considering, on the other hand, the arguments above mentioned in favor of operating, I think we are fairly jnstified in recommending that, not only in traumatic, but in all cases where a young person, one who is under middle age, has cataract in one eye, the lens should be broken up, and removed by absorption."—*Am. Jour. Med. Sci.*, Jan., 1844.

